# Progressive Water Deficit Impairs Soybean Growth, Alters Metabolic Profiles, and Decreases Photosynthetic Efficiency

**DOI:** 10.3390/plants14172615

**Published:** 2025-08-22

**Authors:** Renan Falcioni, Caio Almeida de Oliveira, Nicole Ghinzelli Vedana, Weslei Augusto Mendonça, João Vitor Ferreira Gonçalves, Daiane de Fatima da Silva Haubert, Dheynne Heyre Silva de Matos, Amanda Silveira Reis, Werner Camargos Antunes, Luis Guilherme Teixeira Crusiol, Rubson Natal Ribeiro Sibaldelli, Alexandre Lima Nepomuceno, Norman Neumaier, José Renato Bouças Farias, Renato Herrig Furlanetto, José Alexandre Melo Demattê, Marcos Rafael Nanni

**Affiliations:** 1 Department of Biology, State University of Maringá, Av. Colombo 5790, Maringá 87020-900, Paraná, Brazil; pg406087@uem.br (D.d.F.d.S.H.); wcantunes@uem.br (W.C.A.); 2 Department of Agronomy, State University of Maringá, Av. Colombo 5790, Maringá 87020-900, Paraná, Brazil; pg55482@uem.br (C.A.d.O.); pg405864@uem.br (N.G.V.); pg405896@uem.br (W.A.M.); pg403805@uem.br (J.V.F.G.); ra143742@uem.br (D.H.S.d.M.); asreis@uem.br (A.S.R.); mrnanni@uem.br (M.R.N.); 3 Embrapa Soja (National Soybean Research Center—Brazilian Agricultural Research Corporation), Rodovia Carlos João Strass, s/nº, Londrina 86001-970, Paraná, Brazil; luis.crusiol@colaborador.embrapa.br (L.G.T.C.); rubson.sibaldelli@embrapa.br (R.N.R.S.); alexandre.nepomuceno@embrapa.br (A.L.N.); norman.neumaier@embrapa.br (N.N.); joserenato.farias@embrapa.br (J.R.B.F.); 4 Gulf Coast Research and Education Center, University of Florida, Wimauma, FL 33598, USA; re.herrigfurlane@ufl.edu; 5 Department of Soil Science, Luiz de Queiroz College of Agriculture, University of São Paulo, Av. Pádua Dias 11, Piracicaba 13418-260, São Paulo, Brazil; jamdemat@usp.br

**Keywords:** chlorophyll *a* kinetics, growth and development, JIP test, nonphotochemical quenching, phenomenological modelling, photosynthesis

## Abstract

Soybean (*Glycine max* (L.) Merrill) is highly sensitive to water deficit, particularly during the vegetative phase, when morphological and metabolic plasticity support continued growth and photosynthetic efficiency. We applied eleven water regimes, from full irrigation (W100) to total water withholding (W0), to plants grown under controlled conditions. After 14 days, we quantified morphophysiological, biochemical, leaf optical, gas exchange, and chlorophyll *a* fluorescence traits. Drought induces significant reductions in leaf area, biomass, pigment pools, and photosynthetic rates (*A*, *g*_s_, ΦPSII) while increasing the levels of oxidative stress markers (electrolyte leakage, ROS) and proline accumulation. OJIP transients and JIP test metrics revealed reduced electron-transport efficiency and increased energy dissipation for many parameters under severe stress. Principal component analysis (PCA) clearly separated those treatments. PC1 captured growth and water status variation, whereas PC2 reflected photoprotective adjustments. These data show that progressive drought limits carbon assimilation via coordinated diffusive and biochemical constraints and that the accumulation of proline, phenolics, and lignin is associated with osmotic adjustment, antioxidant buffering, and cell wall reinforcement under stress. The combined use of hyperspectral sensors, gas exchange, chlorophyll fluorescence, and multivariate analyses for phenotyping offers a rapid, nondestructive diagnostic tool for assessing drought severity and the possibility of selecting drought-resistant genotypes and phenotypes in a changing stress environment.

## 1. Introduction

Agriculture in the 21st century faces an unprecedented dual challenge, such as meeting the food demands of a rapidly growing global population. In this sense, safeguarding environmental sustainability and ensuring the resilience of production systems under accelerating climate change are essential. According to FAO projections [1], the global population is expected to surpass 10 billion by 2050, which will demand substantial increases in the production of food, fiber, and renewable energy. Meeting this demand requires not only quantitative improvements in crop productivity but also the adoption of conservation-oriented practices, technological innovations, and the efficient use of natural resources. However, climate change-driven drought events represent one of the most severe threats to global food security, as they directly reduce crop yields, compromise quality, and destabilize agricultural productivity, particularly in regions heavily reliant on rainfed systems.

Brazil, with one of the world’s largest expanses of arable land, has become a global leader in soybean (*Glycine max* (L.) Merrill) production and export, accounting for more than one-third of global trade [2]. In addition to its economic importance, soybean is a major source of high-quality protein, essential amino acids, unsaturated fatty acids, isoflavones, and other bioactive compounds associated with cardiovascular health, a reduced risk of certain cancers, and improved metabolic function [2,3]. States such as Paraná, which contribute approximately 16% of the country’s soybean output, are of strategic importance for both domestic supply and international markets, as well as for the socioeconomic stability of regional agricultural systems. However, soybean productivity is increasingly threatened by climatic variability, particularly drought events, which have intensified in frequency, duration, and severity due to extreme weather patterns and altered rainfall regimes in amplitude [2,3].

Water deficit is widely recognized as one of the most critical abiotic stresses limiting global crop productivity and is responsible for up to 75% of yield losses in major crops [4,5]. In soybeans, drought stress may affect all developmental stages, with especially detrimental impacts during grain filling, resulting in decreases in yield, seed quality, and regenerative potential. Recurring droughts further exacerbate production instability and amplify the economic risks associated with agribusiness operations [6,7,8]. Drought resistance in crops is governed by integrated regulatory mechanisms involving hormonal signaling networks (with abscisic acid as a central regulator interacting with auxin, ethylene, brassinosteroids, and gibberellins), transcriptional control by stress-responsive transcription factors (e.g., DREB, NAC, MYB, and bZIP), activation of antioxidant systems, osmotic adjustment, and anatomical and physiological modifications that sustain water status and photosynthetic efficiency under stress. Understanding these regulatory processes is essential for guiding targeted breeding and management strategies for improving drought resilience [6,7,8,9].

Physiologically, drought stress triggers a complex network of responses, including stomatal closure, reduced photosynthetic capacity, altered hydraulic conductivity, and decreased photosynthesis and water-use efficiency. These responses are often accompanied by metabolic adjustments, such as disrupted membrane integrity and altered secondary metabolism [2,10,11]. Plants exposed to water deficit typically present reduced chlorophyll and carotenoid levels. However, there was increased accumulation of phenolic compounds, lignin, proline, and antioxidant molecules, as well as elevated levels of markers of oxidative stress, such as lipid peroxidation and electrolyte leakage. These responses, which are widely documented in the literature, are acclimation and adaptive mechanisms that help minimize cellular damage and preserve physiological function under stress.

However, the magnitude and nature of these responses depend on the intensity and duration of drought, developmental stage, and genotype, making the development of universal mitigation strategies particularly challenging. In this context, the development of rapid, sensitive, and nondestructive diagnostic tools for early drought detection remains a critical research gap. Therefore, this study aimed to characterize the morphophysiological, biochemical, and photoelectrochemical responses of soybean plants to progressive water deficit during early vegetative growth. Here, progressive drought’ refers to a gradual, controlled reduction in soil water availability over time, simulating the continuous depletion of soil moisture observed under field conditions. We hypothesized that increasing drought severity would induce coordinated structural and functional adjustments physiological and biochemical changes), with identifiable thresholds beyond which photosynthetic performance and carbon assimilation sharply decline. To test this hypothesis, we applied eleven controlled water regimes and assessed multiscale phenotyping metrics, including growth, pigment content, gas exchange, chlorophyll fluorescence, and biochemical markers. Our findings reveal the critical water deficit threshold for maintaining physiological stability and highlight the potential of integrated phenotyping approaches as rapid diagnostic tools for drought stress assessment in soybean. The BRS 1056IPRO cultivar was selected because of its high yield potential and known moderate sensitivity to drought, representing a relevant model for screening early vegetative responses.

## 2. Results

### 2.1. Growth Analysis

**Figure 1:**
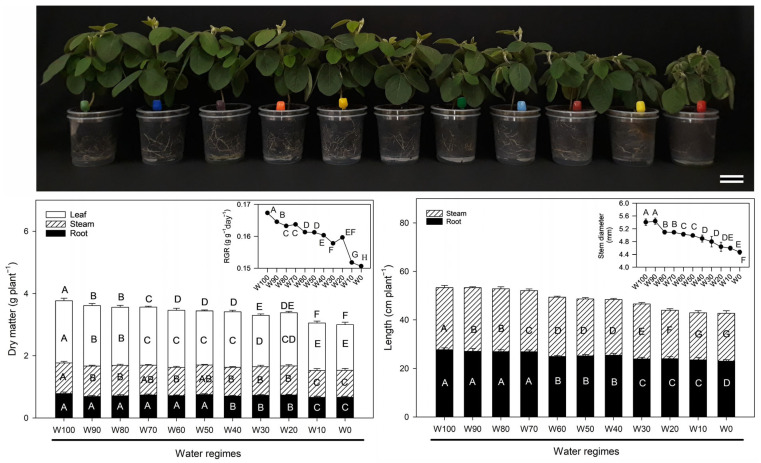
Soybean plants (*Glycine max* (L.) Merrill) were grown under different water regimes 14 days after treatment initiation. The (**upper panel**) shows the visual gradient from fully irrigated plants (W100) to those under progressive water deficit conditions (W90 to W0). The (**lower left**) graph shows the dry matter accumulation (g plant^−1^) in leaves (white), stems (hatched), and roots (black) under each water regime. Letters indicate statistically significant differences among treatments within each plant organ (Fisher’s LSD test, *p* < 0.05). The inset displays the relative growth rate (RGR, g g^−1^ day^−1^) under each treatment, with letters denoting significant differences (Fisher LSD test, *p* < 0.05). The (**lower right**) graph illustrates the total length (cm plant^−1^) of stems (hatched) and roots (black) for each treatment. The inset shows the stem diameter (mm), with different letters indicating significant differences among the water regimes (Fisher’s LSD test, *p* < 0.05). (n = 8 ± SE). Scale bar = 6 cm.

The soybean (*Glycine max* (L.) Merrill) plants clearly presented morphological responses across the eleven water regimes (Figure 1). After 14 days of treatment, the plants maintained under full irrigation (W100–W80) remained vigorous, exhibiting intense green pigmentation, expansive leaf development, and visibly well-developed root systems. As the irrigation level decreased, particularly from W70 to W40, the plants began to show progressive signs of water stress, including moderate reductions in shoot height, leaf area, and chlorophyll intensity.

Under severe water restriction (W30 to W0), drought symptoms became more pronounced. For example, plants were visibly stunted, with evident leaf chlorosis, reduced turgor, leaf folding, and initial wilting observed in the most extreme treatments (W10 and W0). These plants also presented less developed root systems than did those under optimal irrigation.

The total plant dry matter content decreased consistently with increasing water deficit (Figure 1, bottom left). Fully irrigated plants (W100) accumulated the highest biomass (~3.8 g plant^−1^), whereas plants under extreme drought (W10 and W0) presented the lowest values (~2.9 g plant^−1^). Leaf dry matter was significantly greater in W100 and W90 (~2.0 g plant^−1^) than in all the other treatments. A gradual decline was observed from W80 onwards, reaching ~1.3 g plant^−1^ in W0. The stem biomass followed a similar trend, decreasing from ~1.1 g plant^−1^ (W100) to ~0.6 g plant^−1^ (W0). In contrast, root biomass was relatively stable across treatments, with significant reductions observed only under the most extreme deficits (W10 and W0; from ~0.8 g in W100 to ~0.6 g in W0).

Biomass partitioning revealed that water deficit primarily affected the aerial portions of the plant, with the severity of the impact closely aligned with the level of water restriction. Statistically significant differences among treatments are indicated by different letters in the bar charts (Fisher’s LSD, *p* < 0.05).

The relative growth rate (RGR) also declined significantly with increasing drought intensity (Figure 1, bottom left inset). The highest RGRs (~0.17 g g^−1^ day^−1^) were recorded in W100 and W90, whereas the reductions became significant from W60 onwards. The lowest RGR (~0.15 g g^−1^ day^−1^) occurred under W0, indicating substantial inhibition of biomass accumulation.

Water availability also had a strong influence on longitudinal growth and stem diameter (Figure 1, bottom right). The root length remained stable from W100–W80 (~28 cm) but progressively declined from W70, reaching ~23 cm in W10 and ~22 cm in W0. Statistically significant differences in root elongation were detected from W60 onwards. The stem length decreased from ~26 cm (W100–W80) to ~21 cm (W10 and W0), with significant reductions emerging from W60.

The stem diameter followed a similar pattern (Figure 1, bottom right inset), declining from ~5.6 mm in W100 and W90 to ~4.5 mm in W10 and ~4.2 mm in W0. Statistical analysis (Fisher’s LSD, *p* < 0.05) confirmed that progressive drought significantly impaired both stem elongation and thickening.

### 2.2. Morphophysiological Indices

**Figure 2:**
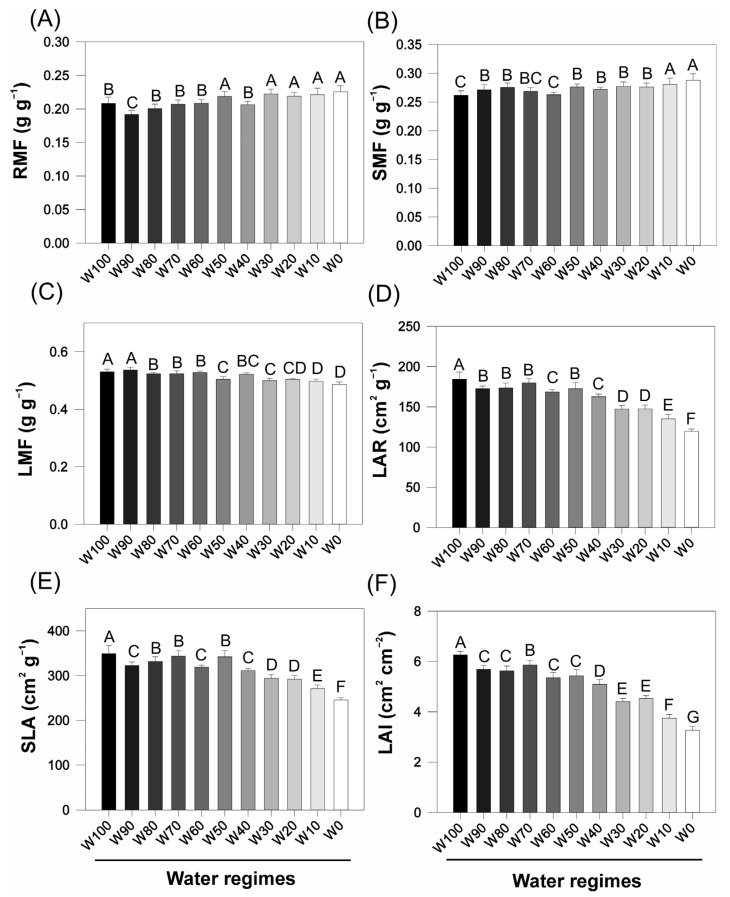
Effects of different water regimes (W100 to W0) on the morphophysiological parameters of soybean plants (*Glycine max* (L.) Merrill) at 14 days after treatment initiation: (**A**) Root mass fraction (RMF). (**B**) Stem mass fraction (SMF). (**C**) Leaf mass fraction (LMF). (**D**) Leaf area ratio (LAR). (**E**) Specific leaf area (SLA). (**F**) Leaf area index (LAI). The bars represent the means ± standard errors. Different letters indicate statistically significant differences among treatments according to Fisher’s LSD test (*p* < 0.05). (n = 8 ± SE).

Water restriction significantly affected the morphophysiological attributes of the soybean plants (Figure 2). The root mass fraction (RMF) increased progressively from 0.21 g g^−1^ in W100 to 0.27 g g^−1^ in W0, with statistically significant differences between the fully irrigated and severely drought-stressed treatments (*p* < 0.05). A moderate increase was also observed in the stem mass fraction (SMF), which rose from 0.28 g g^−1^ (W100) to 0.31 g g^−1^ (W0), although the variation among the treatments was less pronounced in the plants.

In contrast, the leaf mass fraction (LMF) decreased significantly under increasing drought, from 0.51 g g^−1^ in W100 to 0.42 g g^−1^ in W0 (*p* < 0.05), indicating a reduced allocation of assimilates to foliar tissues under water stress. Similarly, the leaf area ratio (LAR) decreased from approximately 175 cm^2^ g^−1^ in W100 to ~115 cm^2^ g^−1^ in W0, reflecting a reduction in the leaf surface area relative to the total plant biomass.

The specific leaf area (SLA) also decreased markedly in response to drought, from ~400 cm^2^ g^−1^ in W100 to ~270 cm^2^ g^−1^ in W0. This reduction suggests potential structural adaptations such as increased leaf thickness under limited water availability. Similarly, the leaf area index (LAI) decreased from 5.8 cm^2^ cm^−2^ in W100 to 3.2 cm^2^ cm^−2^ in W0, indicating a significant loss in canopy development.

Across all indices, statistically significant differences were detected between the well-watered and water-deficit treatments (Fisher’s LSD test, *p* < 0.05), underscoring the pronounced impact of progressive drought on leaf growth and overall canopy architecture in soybean.

### 2.3. Spectral Reflectance Profiles Under Water Regimes

**Figure 3:**
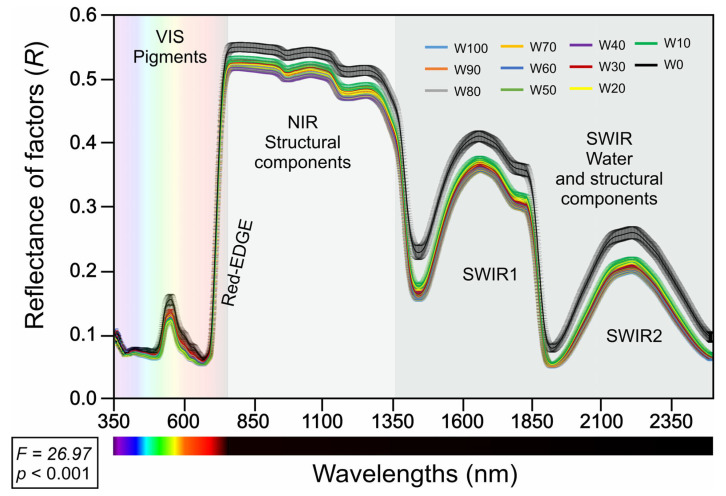
UV–VIS–NIR–SWIR reflectance profiles (350–2500 nm) of fully expanded soybean plants (*Glycine max* (L.) Merrill) leaves under distinct water regimes. Reflectance spectra are shown for all the treatments, ranging from W100 to W0. The spectral domains are segmented as follows: VIS (350–700 nm; pigment absorption), NIR (700–1350 nm; structural leaf properties), and SWIR (1350–2500 nm; water and additional structural components). Significant differences between treatments were detected via one-way ANOVA (F = 26.97, *p* < 0.001). (Mean ± SE). (n = 24 ± SE).

The mean reflectance spectra (350–2500 nm) (Figure 3) of fully expanded *Glycine max* leaves exhibited distinct patterns across the eleven water-regime treatments (W100 to W0). In the VIS region (350–700 nm), all the treatments presented low reflectance values, with clear separation between the regimes: leaves under relatively high water availability (W100, W90, W80) consistently presented relatively low reflectance, whereas those under relatively severe water restriction (notably W30, W20, W10, and especially W0) presented a progressive increase in reflectance, particularly near the red edge (approximately 700 nm).

In the NIR region (700–1350 nm), all the treatments demonstrated a pronounced increase in reflectance. Compared with those under greater water deficit, the treatments with greater water availability (W100 to W60) maintained higher NIR reflectance values, with W0 consistently presenting the lowest NIR reflectance values.

In the SWIR region (1350–2500 nm), two marked absorption features were observed (SWIR1 and SWIR2), with all the treatments resulting in a reduction in reflectance at these wavelengths. The separation among water regimes was most apparent in this domain: the W0 treatment presented the highest reflectance values across SWIR1 and SWIR2, whereas intermediate treatments followed a gradation corresponding to the severity of water deficit.

### 2.4. Biochemical and Physiological Analyses of Leaves, Stems, and Roots

**Figure 4:**
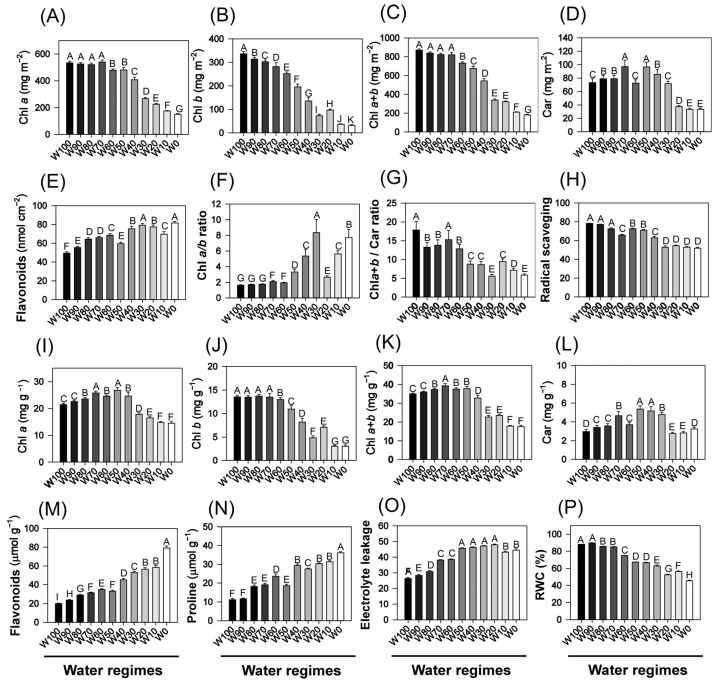
Foliar pigments and physiological parameters of soybean plants (*Glycine max* (L.) Merrill) subjected to different water regimens (W100 to W0) 14 days after treatment initiation expressed as the leaf area and dry weight: (**A**–**C**) Chlorophyll *a*, *b*, and total chlorophyll contents (mg m^−2^). (**D**) Carotenoids (mg m^−2^). (**E**) Flavonoids (nmol cm^−2^). (**F**) Chl *a*/*b* ratio. (**G**) Chl *a* + *b*/Car ratio. (**H**) Radical scavenging activity. (**I**–**K**) Chl *a*, Chl *b*, and Chl *a* + *b* contents on a dry weight basis (mg g^−1^). (**L**) Carotenoids (mg g^−1^). (**M**) Flavonoids (μmol g^−1^). (**N**) Proline (μmol g^−1^). (**O**) Electrolyte leakage (%). (**P**) Relative water content (RWC%). Different letters indicate statistically significant differences between treatments according to Fisher’s LSD test (*p* < 0.05). The means ± SEs are based on three leaflets per sample. (n = 24 ± SE).

Progressive water deficit induced substantial alterations in the foliar pigment composition and physiological status of the soybean plants (Figure 4). The chlorophyll *a* (Chl *a*) content decreased from approximately 800 mg m^−2^ under full irrigation (W100) to ~400 mg m^−2^ under W0. A similar pattern was observed for chlorophyll *b* (Chl *b*), which decreased from ~300 mg m^−2^ to less than 50 mg m^−2^ under severe drought (*p* < 0.05). Consequently, total chlorophyll (Chl *a* + *b*) declined from ~1100 mg m^−2^ in W100 to ~450 mg m^−2^ in W0. The carotenoid (Car) content also significantly decreased from ~110 mg m^−2^ to ~55 mg m^−2^ across the same range of treatments.

The Chl *a*/*b* ratio increased significantly under drought stress, exceeding a value of 7.0 in W0, suggesting the selective degradation of Chl *b* under water limitation. Conversely, the Chl *a* + *b*/Car ratio decreased from ~19 (W100) to ~8 (W0), indicating a proportionally greater reduction in chlorophyll relative to carotenoids, possibly reflecting the greater oxidative stability and protective role of carotenoids. The antioxidant capacity, assessed by radical scavenging activity, also declined progressively with increasing drought severity, with the lowest values recorded in the most water-limited treatments (*p* < 0.05).

When expressed on a dry weight basis, both Chl *a* and Chl *b* decreased gradually across treatments. Under full irrigation, the maximum values were approximately 46 mg g^−1^ and 19 mg g^−1^, respectively, but under severe drought (W0), these values decreased to ~20 mg g^−1^ for Chl *a* and ~4 mg g^−1^ for Chl *b* (Figure 4). The carotenoid content followed a similar pattern, decreasing from ~10 mg g^−1^ in W100 to ~4 mg g^−1^ in W0.

The flavonoid (Flv) content, expressed both per unit leaf area and per unit dry mass, also declined significantly under water restriction, suggesting the suppression of the phenylpropanoid biosynthetic pathway in drought-stressed plants. This reduction may compromise the antioxidant buffering capacity and structural integrity of plants under stress.

In contrast, proline accumulation increased sharply in response to water stress, increasing from ~8 μmol g^−1^ in W100 to ~80 μmol g^−1^ in W0. This trend highlights the central role of proline as an osmoprotectant, contributing to osmotic adjustment, redox balance, and protein/membrane stabilization during drought.

Electrolyte leakage, a sensitive indicator of membrane damage, increased substantially with increasing water deficit, increasing from ~18% in W100 to ~54% in W0. This finding suggests a progressive loss of membrane integrity and increased cellular permeability under stress. Similarly, the relative water content (RWC) declined consistently with increasing drought severity, from ~95% in W100 to ~65% in W0, confirming a significant reduction in tissue hydration and water retention capacity in drought-stressed plants.

### 2.5. Phenolic and Antioxidant Compounds

**Figure 5:**
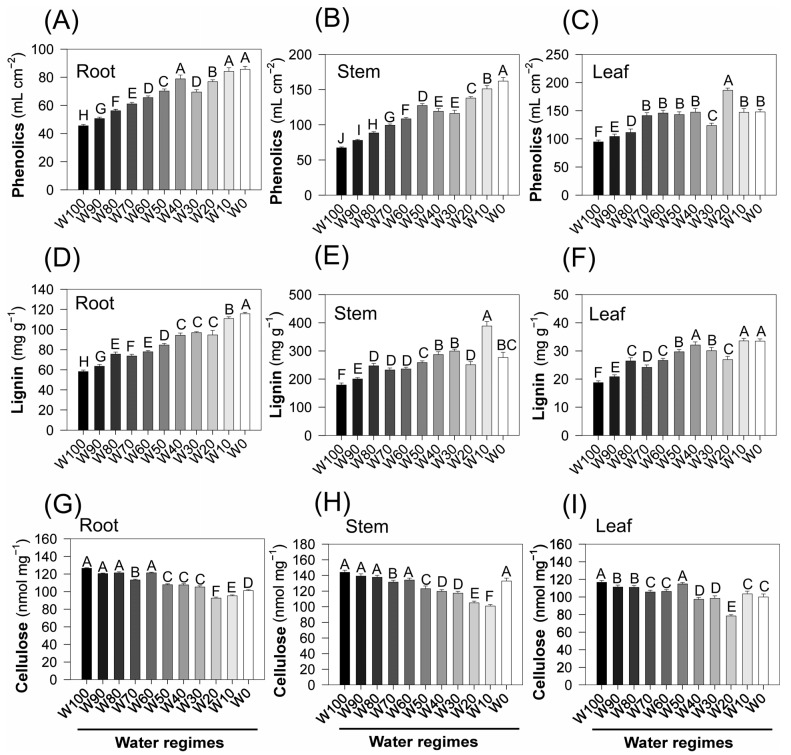
Soluble and structural biochemical components in soybean plants (*Glycine max* (L.) Merrill) subjected to different water regimens (W100 to W0) 14 days after treatment initiation: (**A**–**C**) Phenolic compounds in root, stem, and leaf tissues (mL cm^−2^). (**D**–**F**) Lignin content in root, stem, and leaf tissues (mg g^−1^). (**G**–**I**) Cellulose content in root, stem, and leaf tissues (nmol mg^−1^). Different letters indicate statistically significant differences among treatments according to Fisher’s LSD test (*p* < 0.05). (n = 24), based on three biological replicates per tissue type.

Water deficit induced a marked accumulation of phenolic compounds across all the analyzed organs of the soybean plants (Figure 5). In the roots, the phenolic content increased progressively from approximately 40 mL cm^−2^ under full irrigation (W100) to ~120 mL cm^−2^ under severe drought (W0). A similar pattern was observed in stems, where values rose from ~35 to ~220 mL cm^−2^, and in leaves, from ~80 to ~210 mL cm^−2^. In all the tissues, the increases were statistically significant across the treatments (*p* < 0.05), indicating the active upregulation of phenolic metabolism in response to water limitation.

The lignin content also increased under water stress. In the roots, the values increased from ~60 mg g^−1^ (W100) to ~120 mg g^−1^ (W0). In stems, the increase was even more pronounced, with the lignin content increasing from ~120 mg g^−1^ in W100 to ~400 mg g^−1^ in W0. In leaves, the lignin content increased from ~20 to ~50 mg g^−1^, reflecting cell wall thickening and reinforcement, which are commonly associated with mechanical protection and reduced water loss under drought conditions.

In contrast, the cellulose content decreased as the drought severity increased. In roots, cellulose levels decreased from ~150 to ~110 nmol mg^−1^. Similar reductions were observed in stems (from ~150 to ~80 nmol mg^−1^) and leaves (from ~140 to ~90 nmol mg^−1^), with statistically significant differences among treatments (*p* < 0.05). This reduction likely reflects a downregulation of primary cell wall biosynthesis and a shift in carbon allocation away from extensible structural polysaccharides.

Overall, water restriction promoted coordinated biochemical adjustments across roots, stems, and leaves, characterized by increased accumulation of phenolic metabolites and lignin, coupled with a concurrent reduction in structural carbohydrates such as cellulose. These changes reflect metabolic reallocation from growth-related polymers to defensive and stress-resistant compounds, supporting cell wall rigidity, antioxidant defense, and tissue protection under drought conditions.

### 2.6. Gas Exchange Parameters

**Figure 6:**
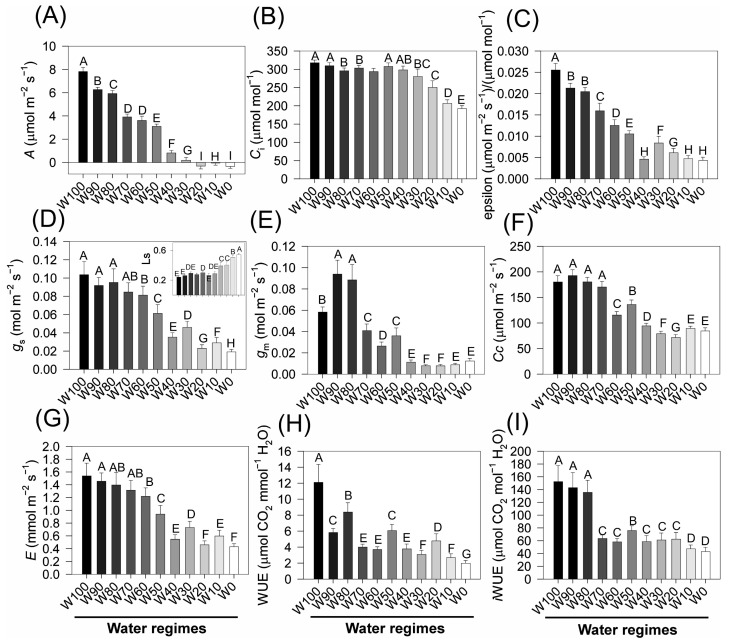
Photosynthetic and gas exchange parameters of soybean plants (*Glycine max* (L.) Merrill) subjected to different water regimes (W100 to W0) at 14 days after treatment initiation: (**A**) Net photosynthetic rate (*A*, µmol m^−2^ s^−1^). (**B**) Intercellular CO_2_ concentration (*C*_i_, µmol mol^−1^). (**C**) Apparent carboxylation efficiency (ε, µmol m^−2^ s^−1^/µmol^−1^ mol^−1^). (**D**) Stomatal conductance (*g*_s_, mol m^−2^ s^−1^). (**E**) Mesophyll conductance (*g*_m_, mol m^−2^ s^−1^). (**F**) CO_2_ concentration in mesophyll cells (Cc, µmol m^−2^ s^−1^). (**G**) Transpiration rate (*E*, mmol m^−2^ s^−1^). (**H**) Water-use efficiency (WUE, µmol CO_2_ mmol^−1^ H_2_O). (**I**) Intrinsic water-use efficiency (iWUE, µmol CO_2_ mol^−1^ H_2_O). Different letters indicate statistically significant differences among treatments (Fisher’s LSD test, *p* < 0.05). The data were derived from three leaflets per sampled plant. (n = 24 ± SE).

Water deficit caused a pronounced reduction in the net photosynthetic rate (*A*), which decreased from approximately 17 µmol m^−2^ s^−1^ under full irrigation (W100) to less than 2 µmol m^−2^ s^−1^ in W0 (Figure 6A), with statistically significant differences among the treatments (*p* < 0.05). The intercellular CO_2_ concentration (*C*_i_) remained above 300 µmol mol^−1^ in W100 but decreased significantly from W50 onwards, reaching ~220 µmol mol^−1^ in W0 (Figure 6B). In parallel, the apparent carboxylation efficiency (ε) decreased progressively from 0.03 to values near 0.008 µmol m^−2^ s^−1^/µmol^−1^ mol^−1^ (Figure 6C), indicating strong inhibition of Rubisco-mediated CO_2_ assimilation under water stress.

Stomatal conductance (*g*_s_) was strongly affected by drought, declining from ~0.13 mol m^−2^ s^−1^ in W100 to <0.02 mol m^−2^ s^−1^ in W0 (Figure 6D). A similar pattern was observed for mesophyll conductance (*g*_m_), which decreased from ~0.10 to <0.02 mol m^−2^ s^−1^ under severe water restriction (Figure 6E). These reductions in diffusive conductance severely limit internal CO_2_ availability.

Consequently, the CO_2_ concentration within mesophyll cells (Cc) decreased substantially, from >200 µmol m^−2^ s^−1^ in W100 to ~60 µmol m^−2^ s^−1^ in W0 (Figure 6F). The transpiration rate (*E*) also decreased sharply, from ~1.8 mmol m^−2^ s^−1^ in W100 to <0.3 mmol m^−2^ s^−1^ under severe drought conditions (Figure 6G), reflecting strong stomatal closure and reduced leaf water loss.

Both water-use efficiency (WUE) and intrinsic water-use efficiency (iWUE) peaked under full irrigation. The WUE reached values near 9 µmol CO_2_ mmol^−1^ H_2_O in W100 but decreased below 2 µmol CO_2_ mmol^−1^ H_2_O in W0 (Figure 5H). Similarly, iWUE declined significantly with increasing drought severity (Figure 5I), underscoring a progressive reduction in carbon assimilation efficiency relative to water use under water-limited conditions.

Together, these results demonstrate that drought stress imposes severe diffusive and biochemical limitations on photosynthesis, characterized by stomatal and mesophyll conductance constraints, the inhibition of Rubisco activity, and a reduction in CO_2_ availability and assimilation. The marked reductions in gas exchange parameters and water-use efficiencies across the water deficit gradient highlight the sensitivity of soybean photosynthetic performance to hydric stress.

### 2.7. Chlorophyll a Fluorescence and Energy Flux Parameters Under Stress

**Figure 7:**
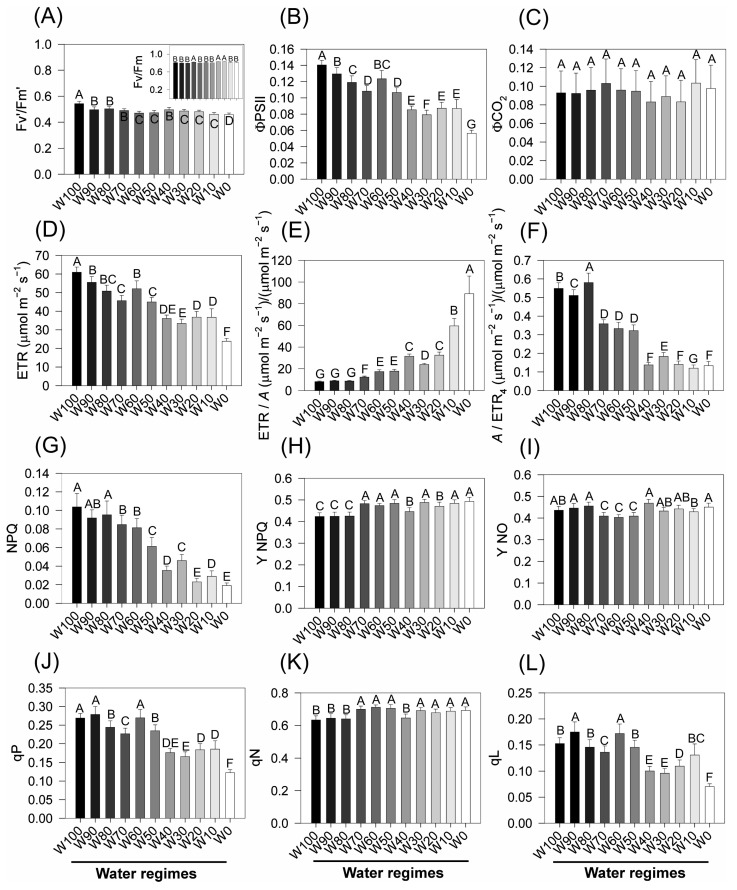
Chlorophyll fluorescence parameters and energy dissipation metrics of soybean plants (*Glycine max* (L.) Merrill) under different water regimes (W100 to W0) 14 days after treatment initiation: (**A**) Maximum quantum efficiency of PSII under light (Fv′/Fm′). (**B**) Effective quantum yield of PSII (ΦPSII). (**C**) Quantum efficiency of CO_2_ assimilation (ΦCO_2_). (**D**) Electron transport rate (ETR). (**E**) Electron transport per unit photosynthesis (ETR/*A*). (**F**) Photosynthesis per unit of electron transport (*A*/ETR_4_). (**G**) Nonphotochemical quenching (NPQ). (**H**) Yield of regulated energy dissipation (Y_NPQ). (**I**) Yield of nonregulated energy dissipation (Y_NO). (**J**) Photochemical quenching (qP). (**K**) Nonphotochemical quenching (qN). (**L**) Coefficient of open PSII centers (qL). Different letters indicate statistically significant differences among treatments (Fisher’s LSD test, *p* < 0.05). The data are based on three leaflets per biological replicate. (n = 24 ± SE).

Significant reductions in the main chlorophyll *a* fluorescence parameters of soybean plants subjected to increasing water deficit were detected after 14 days of treatment (Figure 7). The Fv′/Fm′ ratio (Figure 7A), which indicates the maximum quantum efficiency of PSII under light-adapted conditions, decreased progressively with increasing drought intensity. The plants under full irrigation (W100) presented the highest values (~0.76), whereas those exposed to severe water stress (W0) presented the lowest values (~0.62), with statistically significant differences between extremes (*p* < 0.05).

The effective quantum yield of PSII (ΦPSII) (Figure 7B) followed a similar trend, with values near ~0.15 in W100 and W90, but decreased from W70 onwards, reaching a minimum of ~0.08 in W0. The quantum efficiency of CO_2_ assimilation (ΦCO_2_) (Figure 7C) remained relatively stable (~0.11) under well-watered conditions but decreased significantly in the most restrictive regimes, reaching ~0.08 in W0, indicating diminished carbon fixation efficiency under water limitation.

The electron transport rate (ETR) (Figure 7D) also declined markedly, from ~90 µmol m^−2^ s^−1^ in W100 to ~40 µmol m^−2^ s^−1^ in W0 (*p* < 0.05), reflecting reduced photochemical activity under drought. Notably, the ETR/*A* ratio (Figure 6E) increased considerably, from ~8 in W100 to ~25 in W0, suggesting a progressive decoupling between electron transport and carbon assimilation.

Conversely, the *A*/ETR_4_ ratio (Figure 7F) decreased significantly under stress, from ~0.14 in W100 to ~0.05 in W0, indicating a reduction in the photosynthetic return per unit of electron flow, i.e., decreased biochemical use of excitation energy.

The nonphotochemical quenching (NPQ) index (Figure 7G), which represents regulated energy dissipation as heat, also decreased under drought, particularly in W10 and W0, where values decreased below 0.03, compared with ~0.13 in well-watered plants. The regulated energy dissipation yield (Y_NPQ) (Figure 6H) remained relatively stable across treatments (~0.32–0.36), with slight increases under moderate drought, suggesting an initial capacity to dissipate excess energy effectively.

In contrast, the nonregulated energy dissipation yield (Y_NO) (Figure 7I) increased under drought, increasing from ~0.37 under W100 to ~0.48 under W0. This increase indicates that an increased fraction of excitation energy is lost passively via fluorescence or nonregulated thermal dissipation under severe stress, potentially reflecting PSII overexcitation and photodamage.

Both photochemical quenching (qP) and nonphotochemical quenching (qN) respond to water stress (Figure 7J,K). qP declined substantially, from ~0.33 in W100 to ~0.14 in W0, indicating a decrease in the proportion of open PSII reaction centers available for photochemistry. In contrast, qN showed a modest increase from ~0.60 to ~0.70, suggesting a moderate shift toward alternative energy dissipation pathways.

Finally, the coefficient of open PSII reaction centers (qL) (Figure 7L) decreased drastically under the more severe drought treatments W30, W20, W10, and W0, decreasing from ~0.20 in W100 to ~0.07 in W0. This reduction reveals increased excitation pressure on PSII and a larger fraction of closed centers under stress, further contributing to impaired photochemical efficiency.

### 2.8. OJIP Fluorescence Transients, JIP Test, and Phenomenological Energy Fluxes

**Figure 8:**
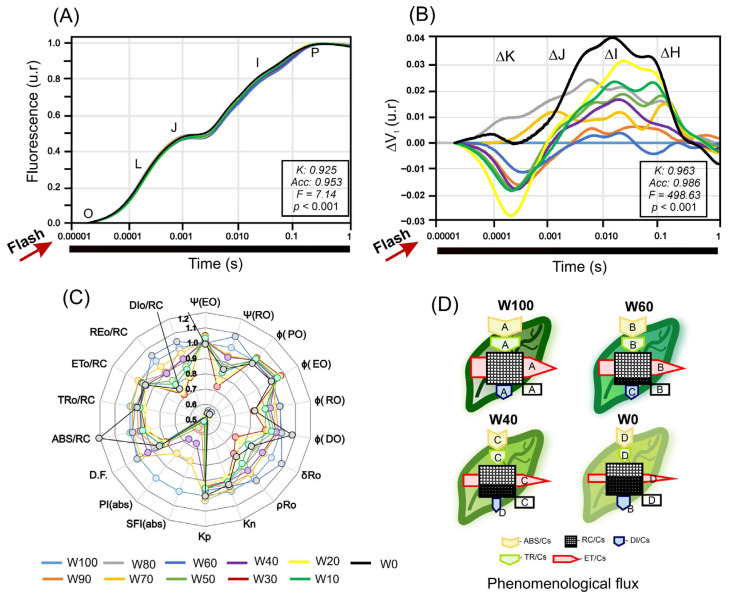
Chlorophyll fluorescence induction kinetics (OJIP curves), JIP test parameters, and phenomenological energy fluxes in soybean plants (*Glycine max* (L.) Merrill) subjected to different water regimes (W100 to W0) 14 days after treatment initiation: (**A**) Average fluorescence transients (OLJIP curve); (**B**) normalized differential kinetics (ΔVt), indicating effects in the ΔK, ΔJ, ΔI, and ΔH phases; (**C**) multiparametric radar plot showing quantum efficiencies [φ(PO), φ(EO)], fluxes per reaction center [ABS/RC, TRo/RC, ETo/RC], and performance indices [PI(abs), SFI(abs), DIo/RC]; (**D**) phenomenological fluxes per cross-section: ABS/Cs (yellow), TR/Cs (green), ET/Cs (red), DI/Cs (blue), and RC/Cs (black grid). Different letters denote statistically significant differences (Fisher’s LSD, *p* < 0.05). The data are based on three leaflets per biological replicate. (n = 24 ± SE).

The average chlorophyll *a* fluorescence induction curves (OJIP) revealed substantial alterations in photochemical dynamics as water deficit intensified in the soybean plants (Figure 8A). The plants maintained under full irrigation (W100 and W90) presented typical OJIP kinetics, with smooth increases across the J–I–P phases, indicating efficient photosynthetic performance and active electron transport. However, from W60 onwards, a pronounced decline in maximum fluorescence levels was observed, and in W0, the P step was notably flattened, reflecting an impaired reduction in plastoquinones and disrupted electron flow through PSII. This flattening of the P phase is consistent with drought-induced photoinhibition and structural damage to PSII.

Differential normalized kinetics (ΔVt) (Figure 8B) highlighted specific changes in the K, J, I, and H steps induced by drought. In the most severely stressed treatment (W0), prominent positive peaks emerged in the J–I–H region, indicating electron accumulation in the plastoquinone pool and a downstream bottleneck in electron transport beyond PSII, a classic hallmark of photoinhibition. These visual patterns were supported by the corresponding JIP test parameters, which showed significant declines in key variables such as φ(EO), ETo/RC, and REo/RC. For example, ETo/RC decreased from 1.00 in W100 to 0.95 in W0, confirming the inhibition of electron flow past Q_A_^−^.

The multiparametric radar plot (Figure 8C) further illustrates these effects. Under well-watered conditions, plants maintained high values for quantum efficiencies of PSII [φ(PO), φ(EO)], fluxes per reaction center [ABS/RC, TRo/RC, ETo/RC], and overall performance indices [SFI(abs), PI(abs)]. However, from W60 onwards, all these indices declined progressively, reaching their lowest levels in W10 and W0. For example, SFI(abs), an index integrating absorption, trapping, and electron transport, decreased by more than 50% under severe drought (~0.45 in W0 vs. 1.0 in W100). Conversely, DIo/RC, which represents the amount of energy dissipated per reaction center, nearly doubled under stress, increasing to ~2.1 in W0.

The phenomenological flux model (Figure 8D) synthesized these alterations at the cross-sectional level, showing consistent reductions in the absorbed energy flux (ABS/Cs), trapped energy flux (TR/Cs), and electron transport flux (ET/Cs) at W60, W40, and W0. In contrast, the energy dissipation flux (DI/Cs) increased significantly, indicating a metabolic shift towards thermal energy dissipation as a photoprotective strategy. Under W0, the lowest values for ET and TR were observed, alongside the highest DI, confirming a functional breakdown of the photochemical apparatus under intense water deficit.

Taken together, these findings demonstrate that drought stress primarily impairs the intermediate phases of the OJIP fluorescence induction curve, severely inhibits the energy fluxes associated with photochemistry, and activates nonphotochemical energy dissipation pathways. These effects were especially pronounced in W10 and W0, where all efficiency, transport, and performance parameters were significantly lower than those in well-irrigated control plants (*p* < 0.05).

### 2.9. Correlation Matrix Analysis

**Figure 9:**
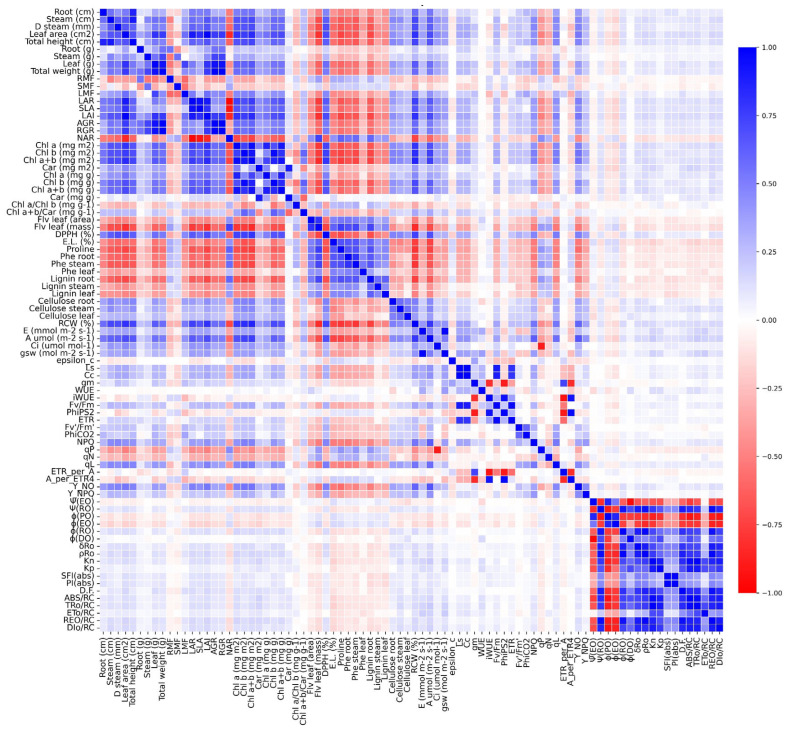
Pearson correlation matrix among morphoanatomical, physiological, biochemical, photochemical, and JIP test variables measured in soybean plants (*Glycine max* (L.) Merrill) subjected to eleven water regimes (W100 to W0). The blue and red colors indicate positive and negative correlations, respectively. The color intensity represents the magnitude of the correlation coefficient (r), with stronger relationships indicated by darker shading. The data correspond to the full dataset (n = 264).

To better understand the integrated responses of the morphological, biochemical, physiological, and photochemical variables under water deficit, a Pearson correlation matrix was constructed (Figure 9). The analysis revealed strong associations among key parameters affected by drought stress.

Photosynthetic variables, including the net photosynthetic rate (*A*), stomatal conductance (*g*_s_), intercellular CO_2_ concentration (*C*_i_), and carboxylation efficiency (ε), were strongly positively correlated (r > 0.85) with leaf pigment contents (Chl *a*, Chl *b*, and Car), biomass accumulation (leaf and total dry mass), and relative water content (RWC). These correlations indicate that carbon assimilation under drought conditions is closely associated with the maintenance of pigment pools and tissue hydration status.

In contrast, the proline content was strongly negatively correlated (r < −0.70) with growth-related traits, including stem diameter, leaf area, specific leaf area (SLA), and total biomass, suggesting that proline accumulation serves as a reliable physiological marker of stress intensity and metabolic reallocation away from growth and toward stress mitigation.

Similarly, both electrolyte leakage and Y_NO, indicators of membrane damage and nonregulated energy dissipation, respectively, displayed strong inverse correlations with photochemical efficiency parameters, such as Fv′/Fm′ and ΦPSII, as well as with quantum yield-related indices. These relationships reinforce the idea that oxidative and structural stress responses are tightly coupled with declines in photosynthetic performance under drought (Figure 9).

Conversely, photochemical performance indices derived from the JIP test, including PI(abs), φ(EO), ETo/RC, and TRo/RC, were positively correlated with each other and with gas exchange parameters but negatively correlated with nonphotochemical dissipation indicators (e.g., DIo/RC, NPQ) and markers of oxidative stress, suggesting an antagonistic dynamic between photochemical efficiency and energy dissipation under increasing stress (Figure 9).

The root phenolic content and lignin accumulation were positively correlated with Y_NO, NPQ, and qN, indicating that metabolic investment in phenylpropanoid-derived compounds is linked with increased energy dissipation and photoprotective mechanisms under drought (Figure 9).

The correlation matrix revealed well-coordinated trade-offs among growth, photochemistry, osmotic adjustment, and antioxidant defense, underscoring the complex and integrated physiological network governing soybean responses to progressive water limitation (Figure 9).

### 2.10. Principal Component Analysis (PCA)

**Figure 10:**
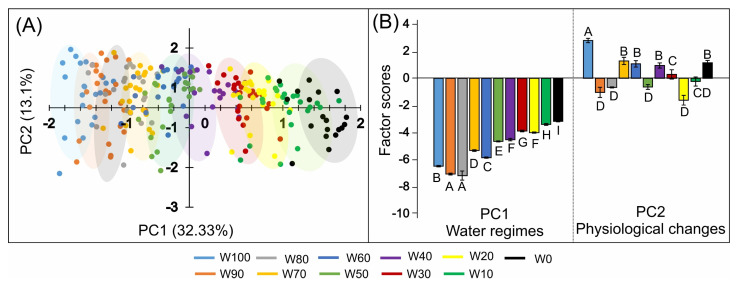
Principal component analysis (PCA) of the morphological, physiological, biochemical, and photochemical traits of soybean plants (*Glycine max* (L.) Merrill) subjected to different water regimes (W100 to W0) 14 days after treatment initiation: (**A**) PCA scatterplot showing individual sample distributions along PC1 and PC2. PC1 separates treatments according to water availability, whereas PC2 captures variation related to physiological adjustments. The ellipses represent 95% confidence intervals. (**B**) Mean factor scores for each treatment on PC1 (water-regime gradient) and PC2 (individual physiological changes). Different letters indicate statistically significant differences among treatments on the basis of Fisher’s LSD test (*p* < 0.05). The data are based on three biological replicates per treatment. (n = 24 ± SE).

Principal component analysis (PCA) revealed a clear multivariate separation of soybean plants in response to the imposed water regimes (Figure 10A). The first principal component (PC1) accounted for 32.33% of the total variance and effectively discriminated treatments along the water availability gradient. The most negative PC1 scores were associated with well-irrigated conditions (W100 and W90), whereas increasingly positive scores corresponded to greater water deficit, particularly under severe drought conditions such as W10 and W0.

The second component (PC2) explained an additional 13.10% of the variance and captured variation related to secondary physiological adjustments under stress. The clustering of individuals by treatment, represented by colored ellipses, showed minimal overlap across water regimes, indicating consistent and distinct physiological profiles associated with each level of water availability.

The group scores presented in Figure 10B support this interpretation. The W100, W90, and W80 treatments resulted in significantly greater negative PC1 scores (ranging from −9 to −7, labelled A and B), reflecting morphophysiological traits typical of well-watered plants. In contrast, the plants subjected to severe drought (W10 and W0) presented the highest PC1 scores (~2.0 to 2.5, labelled H and I), with statistically significant differences across the gradient (*p* < 0.05).

PC2 scores further differentiated specific physiological responses. Particularly in W10 and W0, the values reached ~0.9 and 1.6, respectively, whereas the remaining treatments maintained PC2 values closer to zero or negative. These findings suggest that extremely stressed plants activate distinct metabolic and photoprotective strategies that are not observed in plants grown under moderate or optimal water supply.

PC1 was driven predominantly by morphophysiological traits associated with growth and pigment integrity, underscoring their dominant role in explaining the primary impacts of declining water availability. In contrast, PC2 was shaped almost exclusively by chlorophyll *a* fluorescence parameters, particularly JIP test indices and phenomenological energy fluxes, highlighting the central role of photoprotective and energy-dissipative mechanisms that become prominent once structural limitations are established. Together, the two axes clearly delineate well-watered plants from drought-stressed plants and clarify the physiological shift from growth restriction to photochemical safeguarding as drought intensifies (Table 1).

## 3. Discussion

### 3.1. Phenotypic Growth Alterations

The impact of water deficit on soybean growth and morphology, as observed in this study, reflects a suite of physiological adaptations and survival strategies commonly reported in crop species under drought stress. These responses involve coordinated adjustments in biomass allocation, shoot-to-root balance, and photosynthetic function, which are mediated by hormonal signaling, metabolic shifts, and anatomical modifications (Figure 1 and Figure 2).

Under full irrigation (W100), the plants exhibited vigorous growth, intense green coloration, and well-expanded leaves, which are indicative of optimal cell expansion, chlorophyll biosynthesis, and active photosynthesis [11,12,13,14]. High water availability sustains positive leaf water potential, turgor pressure, and cell elongation, thereby maximizing leaf area and carbon assimilation capacity [15,16].

As water availability decreased (W70 to W40), noticeable reductions in shoot height, leaf area, and pigment content became evident. These symptoms are typically associated with reduced osmotic potential, abscisic acid (ABA)-induced stomatal closure, and limited CO_2_ uptake, ultimately restricting growth and triggering premature senescence under prolonged stress [17,18,19,20].

In severely restricted regimes (W30 to W0), shoot development was strongly inhibited, with visual symptoms including leaf wilting, chlorosis, loss of turgor, and leaf folding. These effects are indicative of accelerated chlorophyll degradation, elevated oxidative stress, and the activation of antioxidant defense mechanisms [21,22]. While moderate drought typically promotes root growth as an adaptive strategy, extreme water limitation (e.g., W0) results in reduced root biomass, suggesting a physiological threshold beyond which the plant prioritizes the maintenance of existing tissues over further exploratory growth [23,24].

Biomass partitioning confirmed that leaves and stems are more sensitive to drought than roots, an adaptive trend that helps reduce transpiration while preserving soil resource acquisition. The observed decrease in the leaf mass fraction (LMF) and increase in the root mass fraction (RMF) support a classical drought response strategy in soybean, as similarly documented in recent studies [25]. In addition, the observed reductions in the leaf area index (LAI) and specific leaf area (SLA) not only reflect decreased leaf surface area but also suggest anatomical adaptations, such as mesophyll thickening and increased lignification, which increase leaf resistance to dehydration and photooxidative damage [26,27,28].

The decline in the relative growth rate (RGR) under water stress, which reflects the efficiency of biomass accumulation per unit mass, reflects two constraints: inhibition of photosynthesis and reallocation of assimilates towards maintenance and stress defense [7,13].

Taken together, these results confirm that drought modulates growth processes at multiple organizational levels, from whole-plant morphology to cellular metabolism. Adaptive responses such as reduced shoot expansion, transient root prioritization, activation of biochemical defenses, and structural reinforcement are key elements of soybean tolerance to water deficit [18,19,29]. These phenotypic adjustments, while initially adaptive, may become limiting under prolonged stress, as reduced shoot development constrains reproductive success and yield potential.

### 3.2. Biochemical and Physiological Alterations

The decline in chlorophyll and carotenoid contents observed under drought stress in this study is consistent with previous findings in soybean and other crop species [30,31]. The reduction in Chl *a* and Chl *b* reflects pigment degradation caused by oxidative stress and nutrient limitations, particularly in nitrogen and magnesium, which are essential cofactors in chlorophyll biosynthesis [32,33,34]. These effects are exacerbated under severe drought conditions (e.g., W0), where excess reactive oxygen species (ROS) compromise chloroplast integrity, resulting in foliar chlorosis (Figure 4).

The decrease in the content of carotenoids, key pigments involved in excess energy dissipation and ROS scavenging, suggests a weakening of the photoprotective system under intense water stress [19,29,35]. The observed increase in the Chl *a*/*b* ratio under drought conditions indicates either selective degradation of Chl *b* or structural reconfiguration of light-harvesting antenna complexes, both of which are well documented adaptive responses in drought-stressed species [36,37,38]. In contrast, the decrease in the Chl (*a*+*b*)/Car ratio highlights the greater vulnerability of chlorophylls relative to the greater oxidative stability of carotenoids.

The reduction in free radical scavenging capacity under severe water deficit further indicates that the plant’s antioxidant defenses may be overwhelmed, likely exceeding the buffering capacity of both enzymatic and nonenzymatic systems [6].

The marked accumulation of proline observed in drought-stressed plants, particularly in W10 and W0, is a well-established physiological hallmark of the drought response in legumes [39,40,41,42]. As both an osmoprotectant and a ROS scavenger, proline contributes to the stabilization of proteins and membranes and helps maintain cellular osmotic homeostasis under dehydration stress [43,44]. Similarly, the observed increase in electrolyte leakage confirms early membrane destabilization, a sensitive indicator of cellular injury induced by drought [45,46].

This is further supported by the decline in relative water content (RWC) under water-limited treatments, reflecting reductions in leaf hydration, turgor pressure, cell expansion, and overall metabolic activity [35,47,48].

The elevated levels of phenolic compounds across roots, stems, and leaves indicate the activation of phenylpropanoid metabolism, a typical biochemical response to abiotic stress [49,50]. These secondary metabolites function as powerful antioxidants and signaling molecules and play important roles in adaptive defense mechanisms under drought conditions [51]. The accumulation of lignin, particularly in stems, reflects cell wall reinforcement, which contributes to reduced transpiration and enhanced mechanical rigidity during dehydration [23,52].

Conversely, the observed reduction in cellulose content across all organs, especially in leaves and stems, may reflect both growth limitations and the downregulation of primary wall biosynthesis, as the plant prioritizes lignin deposition (mechanical stability) over extensibility-related polysaccharides [24].

Taken together, these biochemical responses reveal complex and tightly regulated metabolic reprogramming in soybean under progressive water deficit. The plant mobilizes protective compounds (e.g., proline, phenolics, lignin), downregulates structural carbohydrate synthesis (e.g., cellulose), and causes a loss of critical light-harvesting pigments. These coordinated changes are consistent with the concept of metabolic resilience and represent integrated survival strategies commonly described in drought-sensitive species such as soybean [53,54]. These results align with the known regulatory networks of drought resistance, in which ABA-mediated signaling, transcription factor activation, osmotic balance, and antioxidant protection act synergistically to preserve photosynthetic performance under water deficit [9,53,54].

In addition to serving as stress markers, these metabolites actively contribute to drought tolerance through well-defined physiological mechanisms. Proline accumulation supports osmotic adjustment by lowering the cellular osmotic potential, thus maintaining turgor pressure and water uptake under low soil moisture. Additionally, proline stabilizes proteins and membranes and directly scavenges reactive oxygen species (ROS), limiting oxidative damage. Phenolic compounds, including flavonoids, act as potent antioxidants, neutralizing ROS and reducing lipid peroxidation while also cross-linking cell wall polymers to increase structural rigidity and decrease cuticular transpiration [23,52]. Increased lignin deposition strengthens vascular tissues, preventing xylem vessel collapse and maintaining hydraulic conductivity under tension [9,53,54]. The observed metabolic shift from cellulose biosynthesis toward phenylpropanoid-derived lignin reflects a reallocation of carbon from primary growth to defense-oriented structures. Together, these biochemical adjustments interact with photoprotective mechanisms (e.g., increased energy dissipation and regulated PSII downregulation), forming an integrated protective network that mitigates drought-induced damage and preserves essential physiological functions [2,39,55].

While this study provides a comprehensive phenotypic overview, it is limited by the use of a single genotype and the absence of reproductive-stage assessments [9,56]. Future studies incorporating transcriptomic data or hormone profiling could confirm the proposed mechanistic hypotheses.

### 3.3. Changes in Photosynthetic Activity

The drastic reduction in the net photosynthetic rate (*A*), from ~17 to <2 µmol m^−2^ s^−1^ under drought, reflects the combined influence of stomatal and nonstomatal limitations on CO_2_ assimilation. Initially, the decline in stomatal conductance (*g*_s_) is the dominant factor restricting CO_2_ influx as part of a rapid response to decreasing leaf water potential, which aims to minimize transpirational water loss [2,39,55].

In this study, the reduction in *g*_s_ from 0.13 to <0.02 mol m^−2^ s^−1^ was accompanied by a parallel decrease in the transpiration rate (*E*), a well-documented pattern in drought-stressed plants [57,58]. This stomatal closure caused a significant decline in the intercellular CO_2_ concentration (*C*_i_) from >300 to ~220 µmol mol^−1^, which in turn contributed to a reduction in the mesophyll CO_2_ concentration (Cc) under severe stress (W10, W0).

In addition to stomatal regulation, photosynthetic inhibition is also driven by nonstomatal constraints. Severe water stress led to marked reductions in mesophyll conductance (*g*_m_), carboxylation efficiency (ε), and both instantaneous (WUE) and intrinsic water-use efficiency (iWUE). The decrease in *g*_m_ to values below 0.02 mol m^−2^ s^−1^ in W0 indicates impaired CO_2_ diffusion from intercellular air spaces to chloroplasts, an increasingly recognized limitation under prolonged drought [39,59].

The decline in ε, from 0.03 to ~0.008 µmol m^−2^ s^−1^ µmol^−1^ mol^−1^, reflects biochemical impairments to CO_2_ fixation, most likely associated with Rubisco deactivation, reduced RuBP regeneration, and oxidative damage to Calvin cycle components, such as those mechanisms extensively reported in soybean and other species under water stress [60,61].

The decrease in WUE, from ~9 to <2 µmol CO_2_ mmol^−1^ H_2_O, demonstrates that water deficit not only restricts carbon assimilation but also severely compromises the plant’s capacity to maintain efficient gas exchange. These findings support the notion that drought-adapted plants tend to conserve water at the expense of growth and productivity [55].

Furthermore, the concurrent reductions in iWUE and instantaneous WUE reveal a decoupling between CO_2_ uptake and water loss, likely influenced by anatomical modifications (e.g., cuticle thickening, altered mesophyll cell arrangement), biochemical adaptations (e.g., osmoprotectant accumulation), and stomatal regulation plasticity [40].

Taken together, these findings confirm that photosynthetic performance under drought conditions is constrained by a suite of interaction limitations, including stomatal closure, reduced internal CO_2_ conductance, biochemical inhibition of CO_2_ fixation, and structural leaf adjustments. These mechanisms highlight the physiological trade-offs between preserving water status and maintaining carbon gain under water-limited conditions.

### 3.4. Differential Mechanisms of OJIP Transients, JIP Tests, and Phenomenological Energy Fluxes

The photochemical response of photosystem II (PSII) to drought is complex and multifactorial, affecting every stage from light energy absorption to electron processing in the Calvin–Benson cycle. As illustrated by the OJIP fluorescence curves (Figure 8A), a progressive decline in maximal fluorescence levels became evident from W60 onwards and was dramatically accentuated under severe stress (W0), indicating substantial limitations in the capacity of PSII reaction centers to reduce the plastoquinone pool (Q_A_ and Q_B_) [3,62,63,64].

The flattening of the OJIP curve and the amplification of the ΔK, ΔJ, ΔI, and ΔH signals in the normalized ΔVₜ kinetics (Figure 8B), particularly the J–I–H phase in W0, are classical indicators of electron accumulation and bottlenecks in PSII electron transport. These disruptions may reflect the inhibition of the oxygen-evolving complex (OEC), damage to the D1 reaction center protein, or saturation of Q_A_^−^ reduction [65,66,67]. In agreement with these findings, this alteration is consistent with drought-induced photoinhibition and structural damage to PSII. In particular, it may result from the degradation or inactivation of the D1 protein within the PSII reaction center, which limits electron transfer from Q_A_ to Q_B_, as well as from impairment of the oxygen-evolving complex (OEC), which reduces the donor-side capacity of PSII. Such damage decreases the efficiency of plastoquinone pool reduction and limits downstream electron transport, leading to the characteristic suppression of the P step observed under severe water deficit [64,68,69,70].

These limitations were further confirmed in the JIP test parameters: the quantum efficiency [φ(PO), φ(EO)], flux indices per reaction center [ABS/RC, TR0/RC, ET0/RC], and integrative performance metrics [SFI(abs), PI(abs)] all declined progressively under water deficit (Figure 8C). The reductions in ET0/RC and RE0/RC indicate impaired electron transport beyond Q_A_ and Q_B_, leading to reduced electron delivery to ferredoxin and ultimately decreased regeneration of NADPH and ATP in the Calvin cycle [71,72].

Concurrently, an increase in the energy dissipation flux (DI0/RC) was observed, reflecting the activation of nonphotochemical quenching (NPQ) as a protective mechanism against photooxidative damage. When electron transport is inhibited, excess excitation energy must be safely dissipated, often via xanthophyll-dependent thermal pathways [73]. In W0, DI0/RC approximately doubled relative to W100, confirming that PSII shifted from photochemistry to dissipation under severe drought.

Further evidence of decoupling between photochemistry and carbon assimilation is observed in the reduction in ETR (Figure 7D) and the marked increase in the ETR/*A* ratio (Figure 7E), indicating that electron flux continues despite diminished CO_2_ fixation [74,75,76]. The concurrent decline in *A*/ETR_4_ and increase in Y(NO) support the hypothesis of insufficient photoprotection under extreme stress.

The reductions in key photoefficiency parameters, such as Fv′/Fm′ (Figure 7A), ΦPSII (Figure 7B), ΦCO_2_ (Figure 7C), qP (Figure 7J), and qL (Figure 7L), indicate decreased PSII openness and a reduced capacity to convert excitation energy into effective electron transport. These changes imply an increased prevalence of closed or inactivated PSII centers and reduced photophosphorylation efficiency [77,78]. Interestingly, a slight increase in qN and relatively stable Y(NPQ) under moderate stress suggest that regulated energy dissipation mechanisms are initially sufficient to maintain redox balance but eventually lead to uncontrolled dissipation (Y(NO)) as stress intensifies [79,80].

Collectively, water deficit imposes successive constraints on the photochemical machinery: (i) stomatal closure and limited CO_2_ uptake, (ii) inhibition of electron transfer from PSII (Q_A_–Q_B_ blockage), (iii) decoupling between electron transport and Calvin cycle activity, (iv) activation of NPQ and thermal dissipation pathways, and (v) a decline in PSII reaction center efficiency and energy balance in chloroplasts.

These findings align with previous studies in soybean and other species [35,81] and reinforce that OJIP kinetics and JIP test parameters offer sensitive, integrative diagnostic tools for quantifying the physiological impairment induced by drought stress.

### 3.5. Modulation of Chlorophyll a Fluorescence Responses

Chlorophyll *a* fluorescence and its derived parameters are highly sensitive indicators of the integrity, efficiency, and dynamic adjustment of photosystems under water deficit [65,73,82]. The progressive decline in Fv′/Fm′ observed in soybean plants under increasingly restrictive water regimes in this study directly reflects the reduction in the maximum photochemical efficiency of photosystem II (PSII), a classical marker of photosynthetic stress, under light conditions [71,79,83].

Under optimal conditions (W100), high values of Fv′/Fm′ (~0.76) indicate that most PSII reaction centers are functional and efficiently convert absorbed light into electron transport. As stress intensified (down to W0), this efficiency progressively decreased (~0.62), suggesting that a significant proportion of PSII centers entered a closed or damaged state, as previously reported in soybean [27] and other legumes [84].

The decrease in ΦPSII under drought conditions is associated with the accumulation of excess excitation energy within PSII, increasing the likelihood of reactive oxygen species (ROS) formation and damage to the photosynthetic machinery [82]. The reduced ΦCO_2_ values (Figure 7C) under water-limited conditions further confirm that not only energy dissipation but also effective carbon assimilation is compromised, a phenomenon directly linked to stomatal closure and CO_2_ diffusion limitation [39,45].

The decline in the electron transport rate (ETR, Figure 7D) is consistent with reports for drought-stressed plants, suggesting blockage at key electron transfer sites in the photosynthetic chain [65,67,81]. The increase in the ETR/*A* ratio (Figure 7E) under severe stress highlights a decoupling between electron flow and net CO_2_ assimilation, indicating that a substantial portion of the absorbed light energy is dissipated through alternative, non-assimilatory pathways rather than being channeled into the Calvin cycle [85,86]. This decoupling is a typical response to both diffusive and biochemical limitations imposed by drought.

The reduction in the *A*/ETR_4_ ratio (Figure 7F) confirms the decline in carboxylation efficiency under stress. In parallel, the reduction in NPQ (nonphotochemical quenching) under more severe regimes may appear paradoxical at first glance but has been documented under extreme drought, where regulated energy dissipation mechanisms become insufficient to handle excess energy, leading to increased nonregulated dissipation (Y(NO)) and potential photodamage [39,55].

Indeed, the increase in Y(NO) (Figure 7I) reflects a shift toward unregulated energy loss, which is frequently associated with ROS generation and oxidative damage. Moreover, Y(NPQ) remained relatively stable, suggesting that the regulatory dissipation capacity was exhausted under severe drought. The behavior of qP (photochemical quenching) and qN (nonphotochemical quenching), along with the reduction in qL (fraction of open PSII centers), reinforces that fewer reaction centers remain open under high stress, indicating impaired electron flow and redox cycling within PSII [72,79,83].

Taken together, these data suggest a progressive redirection of energy flow from photochemistry towards dissipation and, ultimately, damage, as the plant loses its capacity to plastically regulate photoprotective mechanisms. This phenomenon is central to the drought and photochemistry literature and has been cited as one of the major physiological constraints on crop productivity under prolonged drought conditions [65,82,87]. Similar responses have also been reported for maize, cotton, canola, and other crops in diverse environments [72,79,83], underscoring the utility of chlorophyll fluorescence parameters as diagnostic tools for stress and for the selection of tolerant genotypes.

### 3.6. Multivariate Analyses in Relation to Total Carbon Gain

The application of principal component analysis (PCA) to integrate physiological, biochemical, and spectral variables represents one of the most powerful tools for understanding systemic plant responses to water deficit [24,26,38]. The clear separation of treatments along PC1 observed in this study reflects the robustness of PCA in capturing the primary effects of the water gradient on soybean growth and physiology. As highlighted in the literature, the variability of drought responses is inherently multifactorial and difficult to detect through univariate metrics alone [24,38]. Multivariate approaches such as PCA enhance the ability to detect latent relationships and multidimensional response patterns.

The dominance of primary and derived growth traits, pigment contents, and stress markers in PC1 (Table 1) demonstrated that, within the 14-day drought exposure window, morphophysiological traits (e.g., leaf area, LAI, RWC, chlorophyll levels, DPPH activity) were the main determinants of carbon assimilation and biomass accumulation. Decreased water potential, combined with reductions in leaf surface area and pigment content, represents the most immediate constraint on plant growth under drought [39,88,89]. Reductions in leaf area, LAI, and pigments directly impact light interception, photosynthetic potential, and water-use efficiency [90].

In contrast, PC2 captured secondary physiological adjustments, particularly photoelectrochemical adaptations and energy fluxes, represented by JIP test parameters and phenomenological flux models. High loadings for ABS/RC, φ(PO), DI0/RC, and Kn indicate that group differentiation under severe drought was driven by fine-tuned regulation of the photosynthetic apparatus, especially increased energy dissipation and reorganization of electron transport [67,72]. This finding reinforces that once structural and pigment limitations are established, survival under intense drought depends on adjustments in quantum efficiency, energy dissipation (NPQ, Y(NO)), and the redox status of PSII reaction centers [73,91].

The importance of biochemical stress markers (proline, electrolyte leakage, phenolics, lignin) in PC1 supports their role as early indicators of oxidative damage and adaptive metabolic responses, as also reported in recent studies on soybean and other legumes [11,84]. In particular, the accumulation of phenolics and lignin is linked to cell wall reinforcement, antioxidant protection, and osmotic homeostasis.

Although yield-related traits were not directly measured in this study, previous research has consistently demonstrated that early vegetative stress responses—such as reduced biomass accumulation, pigment degradation, and decreases in photosynthetic efficiency—are strongly correlated with reductions in reproductive output and final yield in soybean. For example, [92,93,94] reported that sustained decreases in leaf area expansion and carbon assimilation during early growth stages limit the development of reproductive structures, ultimately lowering pod number, seed mass, and total grain yield. Given that plants subjected to water availability below W60 in the present study presented marked impairments in growth, pigment integrity, and photochemical performance, these physiological thresholds are likely to have downstream consequences for reproductive success and yield potential [13,95,96]. These findings underscore the importance of maintaining an optimal water status during early vegetative stages to safeguard both physiological performance and eventual productivity. Nevertheless, as the present study was restricted to the early vegetative stage, caution should be exercised when extrapolating these findings to reproductive development and final yield outcomes.

The integration of multiple biological scales, including morphological, pigmentary, photochemical, and biochemical scales, is crucial for advancing precision phenotyping and selecting drought-tolerant genotypes [2]. This study demonstrated that combining multivariate analysis with spectral and physiological data not only enables precise stress diagnosis but also reveals the predominant adaptive mechanisms operating along the drought gradient.

## 4. Materials and Methods

### 4.1. Plant Materials and Experimental Design

Soybean seeds (*Glycine max* (L.) Merrill) were used in this study. The soybean cultivar BRS 1056IPRO was selected for this study because it is widely cultivated in southern Brazil, particularly in Paraná State, and is recognized for its high yield potential, broad adaptation to regional edaphoclimatic conditions, and stability under commercial production systems. Seeds were first germinated under controlled laboratory conditions. Uniform and vigorous seedlings, free from morphological abnormalities, were selected and transplanted into 1-L plastic pots filled with sterilized substrate.

The experiment was conducted in a growth chamber under strictly controlled environmental conditions: the temperature was maintained at 26 °C during the day and 23 °C at night; the relative humidity was set at 70%; a 16/8 h light/dark photoperiod was applied; and the light intensity was maintained at 500 µmol m^−2^ s^−1^ via an LI-190R (LI-COR Inc., Lincoln, NE, USA) quantum sensor for calibration.

After transplanting, all the seedlings were maintained under full irrigation for seven days to allow acclimation. Following this period, eleven distinct water regimes were established on the basis of substrate field capacity: 100% (W100), 90% (W90), 80% (W80), 70% (W70), 60% (W60), 50% (W50), 40% (W40), 30% (W30), 20% (W20), 10% (W10), and 0% (W0). The water volume was precisely adjusted for each treatment according to the gravimetric soil water content, simulating a gradient of increasing water restriction. Hoagland nutrient solution was applied every two days to ensure adequate nutrient availability throughout the experimental period.

The experimental design was completely randomized, consisting of 11 water regimes and 8 replicates per treatment, for a total of 88 experimental units. All the pots were randomly positioned within the chamber. The water regimes were maintained for 14 consecutive days, during which the environmental parameters and irrigation levels were rigorously monitored and controlled.

### 4.2. Growth Analyses

Vegetative growth parameters were evaluated at 14 days after the onset of the water treatments. A total of 88 soybean plants were used, distributed across 11 water regimes with 8 replicates each, as previously described. On day 0, a subset of plants was reserved to determine the baseline growth parameters.

At each time point, the dry mass of the roots, stems, leaves, and total plant biomass (sum of all the fractions) was determined. The stem diameter, stem length, root length, and total leaf area were also measured. The plant organs were dried at 70 °C in a forced-air oven until a constant weight was reached to ensure accurate biomass determination.

From the primary biomass data, the relative growth rate (RGR) was calculated as $\left[RGR=\frac{\ln\left({\mathrm{DM}}_{2}\right)-\ln\left({\mathrm{DM}}_{1}\right)}{\left(t_{2}-t_{1}\right)}\right]$, where DM1 and DM2 represent the total dry mass at times t1 and t2, respectively [97]. The net assimilation rate (NAR) was computed as $NAR\;=\;\left(\frac{1}{LA}\right)\times \;\left(\frac{\Delta DM}{\Delta T}\right)$, where LA is the total leaf area per plant, and ΔDM is the change in dry mass over the interval Δt. The leaf area index (LAI) was estimated via the model on the basis of ground surface projection $\left[\mathrm{LAI}\;=\;\frac{AF}{\pi \;r^{2}}\right]$, where r is the length of the longest leaf in the plant. Additional derived growth parameters included the leaf area ratio (LAR) ($\left[\mathrm{LAR}=\frac{LA}{{DM}_{\mathrm{Total}}}\right]$), root mass fraction (RMF) $\mathrm{RMF}=\frac{{DM}_{\mathrm{Root}}}{{DM}_{\mathrm{Total}}}$, stem mass fraction (SMF) ($\mathrm{SMF}=\frac{{DM}_{\mathrm{Stem}}}{{DM}_{\mathrm{Total}}}$), leaf mass fraction (LMF) ($\mathrm{LMF}=\frac{{DM}_{\mathrm{Leaf}}}{{DM}_{\mathrm{Total}}}$), and specific leaf area (SLA) ($\mathrm{AFE}=\frac{LA}{{DM}_{\mathrm{Leaf}}}$). The leaf area density (LAD) was calculated by assuming that the aerial portion of the plant formed a conical volume $V_{\mathrm{cone}}=\frac{1}{3}\pi r^{2}h$, where stem height enables estimation of the leaf area per unit volume (cm^2^ leaf/cm^3^ plant).

All growth parameters were determined, allowing comparative analysis across the water regimes [97,98].

### 4.3. Hyperspectral Reflectance Data

Hyperspectral reflectance spectra were obtained from the adaxial surface of fully expanded leaves via a FieldSpec 3 spectroradiometer (ASD Inc., Boulder, CO, USA) equipped with a PlantProbe^®^ leaf clip (ASD Inc., Boulder, CO, USA). The measurements covered the 350–2500 nm spectral range (UV–VIS–NIR–SWIR). Prior to each measurement session, the instrument was calibrated with both a Spectralon^®^ white reference and a dark reference to ensure accuracy. For each leaf, 50 consecutive scans were averaged to reduce noise and improve signal quality. All reflectance data were processed via ViewSpec Pro^®^ software version 6.0 (ASD Inc., Falls Church, VA, USA), following standard protocols for baseline correction and interpolation. Only reflectance values were considered for subsequent statistical and multivariate analyses [52]. All the measurements were conducted under controlled ambient light conditions.

### 4.4. Pigment Profile Extraction

Simultaneous quantification of total chlorophyll (Chl), carotenoids (Car), anthocyanins (AnC), and flavonoids (Flv) in soybean leaf extracts was performed according to the protocols of Gitelson and Solovchenko (2018) [99] and Falcioni et al. (2022) [100], with adaptations for soybean tissues.

Leaf segments (0.5 cm^2^) were homogenized in 1.5 mL microtubes containing a chloroform–methanol extraction solution (2:1, *v*/*v*) in the presence of CaCO_3_ to stabilize pigment integrity. After complete pigment solubilization, distilled water corresponding to 20% of the final extract volume was added to induce phase separation into polar and apolar fractions. The samples were then centrifuged at 15,000 rpm for 5 min to achieve complete separation of the aqueous and organic phases.

Spectrophotometric readings were conducted in 96-well quartz microplates (200 µL per sample) via a Biochrom Asys UVM-340 UV microplate reader ((Biochrome Ltd., Milton Road, Cambridge, UK). Pigment concentrations were calculated on the basis of the absorbance at characteristic wavelengths for each compound group.

#### 4.4.1. Chlorophyll and Carotenoid Quantification

The concentrations of chlorophylls *a*, *b*, *a* + *b*, and carotenoids (carotenes + xanthophylls) were measured by adding 200 µL of methanol extract to each well. The absorbance readings were performed at 470, 652, and 665 nm, and the blank sample was 100% methanol. The concentrations of chlorophylls and carotenoids [Chl*a*, Chl*b*, Chl*a* + *b*, and Car_(C+X)_] were determined via the equations defined by [101] and expressed in terms of leaf area and dry weight:

Chl*a* = 16.72 × Abs665 − 9.16 × Abs652

Chl*b* = 34.09 × Abs652 − 15.28 × Abs665

Chl*a* + *b* = Chl*a* + Chl*b*

Car_(C+X)_ = (1000 × Abs470 − 1.63 × Chl*a* − 104.96 × Chl*b*)/221

#### 4.4.2. Flavonoid and Anthocyanin Quantification

For the quantification of flavonoids (Flv), 400 µL of methanolic extract, 400 µL of chloroform, and 400 µL of water were added to the extract for the separation of the polar phases (upper phase, methanol-water, and extrachloroplastidic pigments) and nonpolar phases (lower phase, chloroform, and chloroplastidic pigments) to avoid contamination of chlorophylls and carotenoids in the readings. The mixture was subsequently centrifuged at 15,000 rpm for 5 min. The upper phase was used for total Flv quantification with a microplate reader at λ358 nm and a molar absorption coefficient of ε358 = 25 mM^−1^ cm^−1^, according to Gitelson and Solovchenko (2018) [99]. After the flavonoids were determined, the water-methanol phase was acidified with hydrochloric acid (HCl; final HCl concentration of 0.1%) and used for AnC quantification at λ530 nm, with a molar absorption coefficient of ε530 = 30 mM^−1^ cm^−1^, according to [99], where 200 µL of the water-methanol phase was added to a 96-well microplate.

#### 4.4.3. Total Soluble Phenolic Compound Quantification in Roots, Stems, and Leaves

Total soluble phenol (PhC) quantification was carried out according to Ragaee (2006) [102], with modifications as described. Phenolic quantification was initiated by the addition of 150 μL of methanolic extract, 70 μL of Folin–Ciocalteu reagent (1 M), 140 μL of Na_2_CO_3_ (3.56 M), and 850 μL of deionized water. The samples were incubated in the dark for 50 min and then centrifuged for 120 s at 15,000 rpm, followed by analysis via a microplate reader at a wavelength of 725 nm.

#### 4.4.4. DPPH-Free Radical Scavenging Activity

To assess antioxidant activity, the free-radical scavenging method using DPPH (2,2-diphenyl-1-picrylhydrazyl) was carried out as described by [103], with modifications. DPPH solution was used at a concentration of 1 mM. The reaction started with the addition of 50 µL of the methanolic extract and 200 µL of the DPPH solution. The samples were shaken and kept in the dark for 60 min. Readings were performed in a 96-well microplate reader at a wavelength of 515 nm [102]. The absorbances obtained were used to calculate the capacity to eliminate free radicals.

% scavenging activity = [1 − (Abs_DPPH_/Abs_sample_) × 100]

AbsDPPH = absorbance of DPPH

Abssample = absorbance DPPH after 60 min

### 4.5. Preparation of Protein-Free Cell Wall Fractions (PFCWs) and Analyses of Lignin and Cellulose

#### 4.5.1. Preparation of Protein-Free Cell Wall Fractions

Protein-free cell wall fractions (PFCWs) were prepared for structural metabolite analysis in soybean roots, stems, and leaves. For each organ, 150 mg of dried, ground plant tissue was weighed into 2 mL microtubes. The samples were subjected to sequential washes to remove soluble compounds: five washes with 50 mM potassium phosphate buffer (pH 7.0), five washes with Triton X-100 (pH 7.0), four washes with 1 M NaCl (pH 7.0), four washes with distilled water, and three washes with acetone. After each step, the samples were centrifuged at 15,000 rpm for 3 min. The final pellets were dried in an oven at 60 °C for 24 h. The resulting material was considered PFCW, which is free of both polar and nonpolar soluble components.

#### 4.5.2. Lignin Determination

The lignin content in the PFCW samples (roots, stems, and leaves) was quantified via the acetyl bromide method. For each sample, 20 mg of PFCW was transferred to a clean microtube, and 0.13 mL of freshly prepared acetyl bromide solution (25% *v*/*v* in glacial acetic acid) was added. The tubes were incubated at 70 °C for 30 min. After digestion, the samples were cooled rapidly on ice, followed by the addition of 0.24 mL of 2 M NaOH, 0.02 mL of 5 M hydroxylamine-HCl, and 1.6 mL of glacial acetic acid to complete lignin solubilization. The tubes were centrifuged at 1400× *g* for 5 min, and the supernatants were used for quantification.

The lignin concentration was determined via a standard curve of alkali lignin and an extinction coefficient (ε) of 22.1 L g^−1^ cm^−1^ at 280 nm. Absorbance readings were taken in a 96-well microplate reader (Biochrom Asys UVM-340) via the ScanPlus VisibleWell^®^ software version 1.0.2 (Biochrome Ltd., Milton Road, Cambridge, UK). The results are expressed as mg lignin per gram of PFCW.

#### 4.5.3. Cellulose Quantification

The cellulose content was quantified following standard protocols adapted for roots, stems, and leaves. The dried plant tissues were incubated at 70 °C for 1 h. After incubation, the ethanol was replaced with an acetic acid/nitric acid mixture for extraction. The samples were subsequently washed with distilled water and treated with freshly prepared anthrone reagent in sulfuric acid. The absorbance was measured at 620 nm via a Biochrom Asys UVM-340 microplate reader (Biochrome Ltd., Milton Road, Cambridge, UK). The cellulose content was expressed as glucose equivalents (µmol glucose g^−1^ dry weight) on the basis of a standard glucose calibration curve.

### 4.6. Gas Exchange and Chlorophyll a Fluorescence Measurements

Integrated gas exchange and chlorophyll *a* fluorescence measurements were performed via a portable open-flow photosynthesis system LI-6800 (LI-COR Inc., Lincoln, NE, USA) equipped with a multiphase flash fluorometer (LI-6800-01). For dark-adapted fluorescence assessments, plants were first subjected to a 12 h dark acclimation period to determine basal fluorescence parameters, including minimum fluorescence (Fo) and maximum fluorescence (Fm). The variable fluorescence (Fv) was calculated as the difference between Fm and Fo, and the Fv/Fm ratio was used as an indicator of the maximum quantum efficiency of photosystem II (PSII) in dark-adapted leaves.

Subsequent measurements were performed on light-acclimated leaves during light-response curve acquisition. The multiphase pulse amplitude modulation protocol was applied by saturating light pulses at 15,000 µmol m^−2^ s^−1^, with a modulation frequency of 5 kHz in darkness and 50 kHz under actinic light. The maximum fluorescence under light (Fm′) was measured at 250 kHz during the saturating pulse, with fluorescence emission detected above 700 nm.

Gas exchange measurements were conducted simultaneously, allowing the determination of physiological parameters such as the net CO_2_ assimilation rate (*A*, µmol m^−2^ s^−1^), stomatal conductance (*g*_s_), transpiration rate (*E*), and water-use efficiency (WUE), as automatically computed via LI-COR software (version 1.0).

In parallel, light-acclimated fluorescence parameters, including the maximum quantum efficiency under light (Fv′/Fm′), effective quantum yield of PSII (ΦPSII), apparent quantum yield for CO_2_ assimilation (ΦCO_2_), electron transport rate (ETR), nonphotochemical quenching (NPQ), photochemical quenching (qP), and nonphotochemical quenching component (qN), were recorded. These parameters provide insight into the functionality and photoprotective responses of PSII under different water regimes.

### 4.7. Fluorescence OJIP Data Collection

The chlorophyll *a* fluorescence kinetics spectrum was measured via an LI-6800 LICOR (IRGA; LI-6800, LICOR, USA; Gax Exchange System) on leaves that were acclimated for 60 min in a dark, humid chamber. Fluorescence curves were obtained using the following settings: sample chamber (6 cm^2^), relative humidity (75%), CO_2_ (400 ppm), fan speed (10,000 rpm), pulse of saturating light (625 nm) of 15,000 µmol m^−2^ s^−1^ for 1 s, and 500 Hz output rate in induction mode. Relative fluorescence intensity was measured at 20 µs, 50 µs, 100 µs, 300 µs, 2 ms, 30 ms, and Fm_t0-tf_ to perform the JIP-test between 20 µs and 1 s. The curves were normalized as variable fluorescence (ΔVt), and the difference in kinetics for each OLJIP phase was calculated with green leaves (L01) as a reference. The top four bands, ΔK (at ~300 µs), ΔJ (at ~2 ms), ΔI (at ~10 ms), and ΔH (at ~40 ms), were calculated, resulting in 925 points of high resolution. The JIP test parameters were calculated according to Strasser et al. (2004) [104].

### 4.8. Statistical Analysis

#### 4.8.1. Univariate Statistical Analysis

Statistical analyses of agronomic, physiological, biochemical, and spectral data included the computation of means, standard error of the mean (SEM), maximum and minimum values, and coefficient of variation (CV, %). Differences among treatment means were assessed by one-way analysis of variance (ANOVA), with statistical significance set at *p* < 0.05. When significant effects were detected, multiple comparisons were performed via Fisher’s LSD (*p* < 0.05). To evaluate the relationships among physiological parameters, vegetation indices, and growth traits, Pearson’s correlation analysis was employed. All univariate statistical analyses were conducted via Statistica 10^®^ software (StatSoft Inc., Tulsa, OK, USA). Graphical data visualizations were generated via SigmaPlot 10.0^®^ (Systat Inc., Santa Clara, CA, USA) and CorelDraw 2020^®^ (Corel Corporation, Ottawa, ON, Canada).

#### 4.8.2. Multivariate Statistical Analysis

Principal component analysis (PCA) was performed on selected physiological and spectral datasets to reduce dimensionality and identify major groupings and response patterns associated with water-regime treatments. The optimal number of principal components was determined on the basis of the criterion of the maximum cumulative variance explained. All multivariate analyses were carried out via The Unscrambler X version 10.4 (CAMO Software, Oslo, Norway), with statistical significance set at *p* < 0.05.

## 5. Conclusions

Progressive water deficit in soybean triggers an integrated sequence of morphological, physiological, and biochemical changes that collectively reduce plant growth, pigment integrity, and photosynthetic performance. While well-watered plants sustain high productivity and physiological stability, plants subjected to mild-to-moderate deficits for up to 14 days (e.g., W100–W70) still maintained relatively stable growth, pigment levels, and photosynthetic activity, indicating a short-term tolerance window before major declines occurred. Water restriction (W60 to W0) beyond this threshold leads to decreased biomass and photosynthetic efficiency, intensified oxidative stress, and structural cell wall modifications. Multivariate analysis revealed two key adaptive domains: growth and pigment maintenance under adequate water supply and photoprotective adjustments under severe stress. This threshold is agronomically relevant, as it indicates an irrigation window that can prevent irreversible yield losses under progressive drought. These findings define physiological thresholds beyond which water scarcity severely limits carbon assimilation and highlight the value of chlorophyll fluorescence and multiscale phenotyping as effective tools for monitoring and characterizing progressive water stress in soybean.

## Figures and Tables

**Table 1:** Percent contribution of the eight functional-trait groups to the first two principal components extracted from the PCA of soybean responses to the 11 water-regime treatments. PC1 represents the drought (water availability) gradient, whereas PC2 reflects secondary physiological adjustments under stress. The percentages within each column sum to 100%.

Groups	PC1—Water Regimes (%)	PC2—Physiological Changes (%)
Primary growth	21.44	0.26
Derivate growth	19.97	0.38
Pigments	19.29	0.76
Stress marker	19.39	0.38
Photosynthetic parameters	10.56	0.21
Fluorescence parameters	3.78	0.43
JIP-test	2.78	46.56
Phenomenological fluxes	2.78	51.03
**Total**	**100**	**100**

## Data Availability

The original contributions presented in this study are included in the article. Further inquiries can be directed to the corresponding author.
